# The opportunities and challenges of working with the development of the ellipse-project before, during and after the COVID-19 pandemic

**DOI:** 10.1192/j.eurpsy.2021.190

**Published:** 2021-08-13

**Authors:** A. Baran

**Affiliations:** Dept. Of Psychiatry, Blekinge Hospital, Karlshamn, Sweden

**Keywords:** COVID-19, educational program, Suicide prevention, students

## Abstract

COVID-19 pandemics has influenced many research and educational projects, and it influenced also ELLIPSE-project, aiming at the creation of a free online curriculum in suicide prevention for students in the higher education in Sweden, Austria, Hungary, Norway and Poland (September 2019-August 2022). The ELLIPSE (E-Lifelong Learning In Prevention of Suicide in Europe) (www.e-llipse.com) is the first international project in the area of education in suicide prevention in the higher education co-founded by the European Union Erasmus+ programme. We will reflect on some opportunities and challenges due to COVID-19 pandemics. Identified opportunities: 1) learning a new way of communicating, 2) reaching more participants (in case of Sweden we were able to get participants from more regions, 3) more frequent meetings heightening the quality of the project, 4) travelling less (online is the more eco-friendly solution). But there were also some challenges: 1) not be able to proceed according to the preliminary plan generated much more administrative work, 2) online meetings was a challenge for those partners who had problems adjusting to the online form of communication, 3) more negative attitudes to the canal of communication resulted in avoiding it, leading to less frequent interactions what resulted in diminished motivation to the project, and being unable to proceed with project tasks, 4) resignation of some patients from participation because of difficulties in accepting the online form of communication. We will discuss these issues in a more detailed way during the interactive workshop.

**Disclosure:**

The E-Lifelong Learning In Prevention of Suicide In Europe (ELLIPSE)-project is co-funded by the European Union’s Programme Erasmus+ (Project ID: 2019-1-SE01-KA203-060571). The EU Commission’s support for this project does not mean that the Commission end

